# Correction: Noninvasive target CT detection and anti-inflammation of MRSA pneumonia with theranostic silver loaded mesoporous silica

**DOI:** 10.1039/d0ra90030c

**Published:** 2020-04-17

**Authors:** Hao Zhang, Qingqing Ding, Jing Ding

**Affiliations:** Department of Geriatric Gastroenterology, The First Affiliated Hospital with Nanjing Medical University Nanjing People’s Republic of China; Department of Respiratory Medicine, The Affiliated Nanjing Children Hospital with Nanjing Medical University Nanjing 210008 People’s Republic of China djnjch@sina.com +86-25-83304239 +86-25-83117319

## Abstract

Correction for ‘Noninvasive target CT detection and anti-inflammation of MRSA pneumonia with theranostic silver loaded mesoporous silica’ by Hao Zhang *et al.*, *RSC Adv.*, 2016, **6**, 5049–5056.

The authors regret that an incorrect version of Fig. 1 was included in the original article. The correct version of Fig. 1 is presented below.

**Fig. 1:**
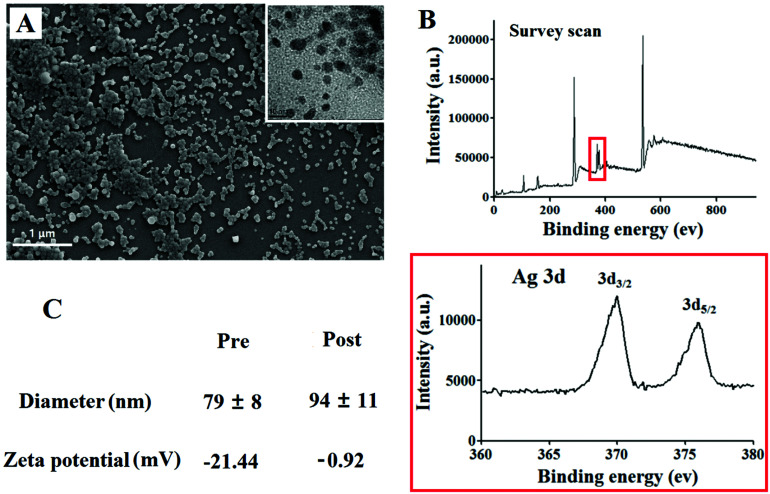
(A) SEM image of PEGylated SLS NPs; inset: high-resolution TEM image highlighting the anchored Ag NPs. (B) XPS result of the SLS NPs and silver element. (C) DLS and zeta-potential profiles of the SLS NPs pre- and post-PEGylation.

The Royal Society of Chemistry apologises for these errors and any consequent inconvenience to authors and readers.

## Supplementary Material

